# Oxygen Inhalations Should Be Warmed

**Published:** 1908-01-18

**Authors:** 


### OXYGEN INHALATIONS SHOULD BE WARMED.

The question of whether oxygen inhalations are beneficial or not in cases such as lobar-pneumonia, pericarditis, broncho-pneumonia, and so forth, is open to discussion, though many of those who have seen their administration in a large number of cases hold the opinion that there are instances in which life is saved thereby.

It seems clear that the action of the oxygen cannot be to make the arterial blood more saturated with that gas; it appears to be rather a direct stimulant to the heart and lungs. We do not propose to discuss here the way in which it acts, nor the comparative inefficacy of the funnel method of its administration. We wish to lay stress upon one single point, namely the extreme coldness of the gas as it leaves the cylinder, and therefore its coldness when inhaled without being warmed *en route* to the patient.

The sudden release of gas that is under extreme pressure leads to a very great fall in the temperature of that gas. The method is used commercially—the liberation of gas from $CO_2$ liquefied under pressure is a common process for freezing things. When an anæsthetist gives a patient gas, the iron bottle from which the nitrous oxide comes is soon coated with a white dew, deposited upon it from the air as the cylinder becomes colder and colder. The same coldness occurs in the case of the oxygen cylinder, and yet again and again one sees the oxygen, colder than the atmosphere of the room, administered to patients with pneumonia or other lung trouble without any attempt being made to warm the gas before it is breathed. The ill effects of very cold inhalations in such cases are perhaps difficult to prove; but no doctor would allow a patient with pneumonia to breathe a cold east wind if he could help it. He ought not to allow the breathing in of this cold oxygen either.

The remedy is very simple. If, instead of having only a foot or two of tubing between the nozzle of the oxygen cylinder and the funnel or face-mask, the connecting tubing is made six or eight feet long, its central portion can be coiled either once or twice, and the coils may be immersed in a basin of warm or hot water. The oxygen as it passes through the tubing then has the chill taken off it very quickly, and the patient will receive all the benefits of the oxygen itself without any of the ill effects that may result from its coldness as administered in many cases

